# ‘Is there any point in me doing this?’ Views and experiences of women in the Diabetes and Antenatal Milk Expressing (DAME) trial

**DOI:** 10.1111/mcn.13307

**Published:** 2021-12-22

**Authors:** Anita M. Moorhead, Lisa H. Amir, Della A. Forster, Sharinne B. Crawford

**Affiliations:** ^1^ Judith Lumley Centre, School of Nursing and Midwifery La Trobe University Bundoora Victoria Australia; ^2^ Midwifery and Maternity Services Research Royal Women's Hospital Parkville Victoria Australia

**Keywords:** antenatal expressing experience, breast milk, breastfeeding, diabetes, pregnancy, qualitative research

## Abstract

The Diabetes and Antenatal Milk Expressing (DAME) randomised controlled trial (RCT) was conducted in 2011–2015, at six sites in Melbourne, Australia to explore the effect of advising women with diabetes in pregnancy to express breast milk from 36 weeks gestation. Infants whose mothers were randomised to express in pregnancy were more likely to be exclusively breast milk fed during their hospital stay, and there was no evidence of harm. This paper explores women's views and experiences of antenatal expressing. In this two‐arm RCT, 635 women with diabetes in pregnancy who were otherwise of low medical risk were randomised at 36–37 weeks gestation to usual care (not expressing, *n* = 316), or the intervention, where women were advised to hand express for 10 min twice daily until birth (*n* = 319). Semistructured face‐to‐face interviews were conducted with 10 women who expressed antenatally. They were asked about their experiences of antenatal expressing, including how they felt about the overall experience, the amount of breast milk they expressed, making time to express, and their experience of breastfeeding. Thematic analysis of the in‐depth interviews identified six themes: (1) learning and adapting expressing, (2) feelings and sensations associated with expressing, (3) support, (4) dis/empowerment, (5) health, and (6) the value of breast milk. Women had both positive and negative experiences of antenatal expressing. If health professionals are advising antenatal expressing to women, it is important they understand the range of outcomes and experiences.

## Key messages

- Women's experiences of antenatal expressing were both positive and negative; it is important to understand the range of experiences.
- Women should be advised that some women express little or no breast milk antenatally and therefore be reassured if they are concerned.
- For women with diabetes in pregnancy, their diabetes management is a significant time burden, and should be considered when discussing antenatal expressing with women.
- Maternity care providers need to follow‐up with pregnant women advised to express, to provide education, reassurance, and reassess expressing techniques.
- Maternity care providers need to manage and prioritise the use of expressed breast milk.

## 1 INTRODUCTION

The World Health Organization (2017) recommends breastfeeding for 6 months exclusively and continued breastfeeding combined with solid foods for up to 2 years or beyond. However, for some women this is more challenging. Women with diabetes in pregnancy—which includes 13% of pregnant women in Australia (Australian Institute for Health and Welfare, 2020)—and their infants are at increased risk of complications during pregnancy and birth (HAPO Study Cooperative Research Group et al., 2008). These complications can be a barrier to successful breastfeeding (Patil et al., 2020). Women with diabetes in pregnancy are at increased risk of delayed lactogenesis II (De Bortoli & Amir, 2016), are less likely to breastfeed exclusively (Oza‐Frank et al., 2016), and have a shorter duration of breastfeeding (Nguyen et al., 2019). However, for women with gestational diabetes mellitus (GDM), breastfeeding confers significant maternal metabolic benefits and delays the possible onset of type 2 diabetes (Aune et al., 2014).

Infants of women with diabetes in pregnancy have a high risk of hypoglycaemia (Mitanchez et al., 2015), which along with the possible maternal delayed lactogenesis II (De Bortoli & Amir, 2016), leads to mother‐infant separation, infant formula supplementation and shorter breastfeeding duration (Baerug et al., 2018). As a result, many pregnant women with diabetes are being advised to express breast milk in the last weeks of pregnancy, to have a supply of expressed breast milk (EBM) to prevent or treat neonatal hypoglycaemia, and avoid infant formula and/or admission of baby to a neonatal unit (NHS Worchestershire Acute Hospitals, 2019; Women's Health Auckland, 2019).

Postnatal expressing is common when women are separated from their babies, when they return to paid employment (Johns et al., 2013), or if babies are unable to feed directly from the breast (Keim et al., 2017). The research on methods of milk expression for lactating women is predominantly breast pump industry‐funded and focused on the amount and content of breast milk, and speed and ease of expressing (Becker et al., 2016). A systematic review of the prevalence and outcomes of expressing by Johns et al. (2013) found limited research about women's experience of expressing.

More recently, studies of antenatal expressing in Australia (Brisbane & Giglia, 2015; Casey, Mogg, et al., 2019; Forster et al., 2011), the United Kingdom (Fair et al., 2018), and the United States (Demirci et al., 2019) have included some exploration of women's experiences. Positive experiences include feelings of increased breastfeeding preparedness or confidence, and that having breast milk provided a sense of security or satisfaction (Brisbane & Giglia, 2015; Demirci et al., 2019; Forster et al., 2011). Negative experiences include feelings of breast tenderness or pain, difficulty with expressing (Brisbane & Giglia, 2015; Demirci et al., 2019; Forster et al., 2011), and embarrassment and concerns about privacy (Brisbane & Giglia, 2015; Demirci et al., 2019). Other experiences include feelings of futility (Brisbane & Giglia, 2015; Casey, Mogg, et al., 2019) and pressure to succeed (Casey, Banks, et al., 2019), feeling anxious about not getting ‘enough’ breast milk, and difficulty finding time to express (Forster et al., 2011). Practical issues were identified, including lack of storage space, wastage of EBM (Casey, Mogg, et al., 2019; Demirci et al., 2019), and perceived lack of support by hospital staff (Demirci et al., 2019).

The Diabetes and Antenatal Milk Expressing (DAME) multisite randomised controlled trial conducted in Melbourne, Australia between 2011 and 2016, tested the practice of advising women with diabetes in pregnancy, who were otherwise of low obstetric risk, to express in pregnancy. While all 635 participants had routine antenatal care, following randomisation, half of the women were verbally guided how to express breastmilk by the research midwives at the hospitals. They were advised to hand express twice a day for no more than 10 min each time and given written instructions about expressing and breastmilk storage as per Supporting Information File 1 of the trial protocol paper (Forster et al., 2014). Infants whose mothers were randomised to express in pregnancy were more likely to be exclusively breast milk fed during their hospital stay, and there was no evidence of harm. Women in the intervention arm expressed a median of 20 times (range: 1–59 times), with the median total volume expressed 5.5 ml (range: 0–905 ml), and almost a quarter of women expressed less than 1 ml in total (Forster et al., 2017).

In follow‐up surveys of all participants the DAME trial at 2 and 12 weeks after birth, many women commented on the low volumes of breast milk they had been able to express. Given their comments, the low median volumes identified, and that perceived insufficient milk supply remains one of the most common reasons cited for ceasing breastfeeding (Hauck et al., 2011), it is important to explore women's experiences of antenatal expressing, including their views of the volume of milk expressed. The purpose of this study was to understand the views and experiences of antenatal milk expressing for a sample of women in the intervention arm of the DAME trial.

## 2 METHODS

### 2.1 Study design

A qualitative descriptive study was undertaken. Our aim was to describe a broad range of experiences and to stay close to the words and meaning of the women (Sandelowski, 2000). To account for diversity, and to maximise the richness of data about participants' views and experiences, we used purposive sampling (Sandelowski, 2000). We considered women's diabetes type (either pre‐existing or pregnancy induced), parity (first baby or not), and volume of breast milk expressed (either higher or lower than 5.5 ml in total). Informed by these criteria, 15 of the most recent participants from two of the largest study sites, were telephoned and invited to participate in an in‐depth interview about their experience in the trial. Ten women consented to participate.

A semistructured interview guide was developed, informed by open‐ended responses to telephone surveys of participants in the trial. The guide was piloted with researchers who were experienced with in‐depth interviews and/or breastfeeding research. Feedback was incorporated, and the guide piloted for understanding and acceptability with a mother known to have recently expressed antenatally. The guide was used to explore participants' overall experience of antenatal expressing, but also included prompt questions about specific aspects of expressing. Women were also encouraged to discuss any aspect of antenatal expressing important to them. Being an iterative process, two questions were added after the first three interviews—one about using a breast pump antenatally, and one exploring participants' impressions of what their family or friends thought about their expressing (Table 1).

**Table 1:** Semistructured interview guide

Key domains
General experience of expressing antenatally—including process, instructions, equipment
Making the time to express
What was noticed while expressing for mother and baby/foetus—sensations, contractions
How women felt about the amount of breast milk that was able to be expressed antenatally—importance, volumes
The impact of expressing antenatally on the postnatal experience—confidence, feeding, expressing
Whether participating in the trial made a difference for women and their babies postnatally—blood glucose management, formula use
Thoughts of partner/family members about antenatal expressing
Other thoughts—intention to express antenatally again, aspects of expressing to change, advice to researchers

### 2.2 Data collection

Interviews were conducted between March and December 2017, at which time the women's babies were between 17 and 47 months of age (median 24 months). A plain language statement and consent form were emailed to women and a consent form signed before interview. Participants were interviewed face‐to‐face at a time and place suitable for them, such as their home, shopping centre or workplace. One interview was conducted by video conference. All interviews were conducted by the lead author. The research team reviewed the data after the third and ninth interviews. No new ideas were elicited from the tenth participant, so we did not seek further women to interview. All interviews were audio recorded, transcribed verbatim and field notes were taken by the researcher at the time of interview. The average length of interview was 36 min (range: 19–53 min).

### 2.3 Analysis

Thematic analysis was conducted to identify, analyse, and generate themes from the data (Attride‐Stirling, 2001; Sandelowski, 2000). The process of inductive coding was used, to stay close to intent of the thoughts and words of the participants (Braun & Clarke, 2006; Saldana, 2016). All transcripts were read and checked against the audio files and reread for understanding and immersion in the data. Initially, three transcripts were independently coded by authors A.M., L.A. and S.C. and were reviewed and discussed for consistency of code identification. A further two transcripts were coded by A.M. and S.C. to confirm coding consistency and the remaining transcripts were coded by A.M. All the transcribed verbatim interview data were given codes and were then organised into broader categories and themes. During the identification and development of the codes and categories, there was extensive discussion between the primary coder and other research members. Where required, the research team reviewed the original transcripts, researcher field notes and some audio‐recordings to check meaning and facilitate agreement. Finally, all categories, themes and interpretation of the data were discussed until agreement was reached. These processes helped ensure that our interpretation reflected the intention of the participants and reduced potential researcher bias. The Standards for Reporting Qualitative Research (SRQR) guidelines were followed to ensure rigour in the conduct and reporting of the research (O'Brien et al., 2014).

The use of direct quotes used in each theme are verbatim, with the emphasis and sentence structure reflecting participants' voices. To protect the confidentiality and anonymity of the participants each woman was assigned a number (e.g., P1).

### 2.4 Ethics

Ethical approval for the DAME study was granted by La Trobe University (11‐004), the Royal Women's Hospital (RWH) (HREC‐11/07) and the Mercy Hospital for Women (MHW) (HREC‐R11/06).

## 3 RESULTS

Ten women participated in an interview. Their characteristics are presented in Table 2.

**Table 2:** Characteristics of participants

Participant	Identifier^a^	Age range at interview (years)	First baby (yes/no)	Age of baby at interview (months)	Country of birth	Type of diabetes during pregnancy	Number of times expressed	Total volume expressed
**1**	[P1, GDM, low]	30–34	No	17	Ireland	Gestational—diet controlled	27	Nil
**2**	[P2, GDM‐in, low]	35–39	Yes	17	Indonesia	Gestational—requiring insulin	16	Nil
**3**	[P3,T2, low]	30–34	Yes	26	India	Type 2 diabetes	11	2 ml
**4**	[P4, GDM, high]	30–34	No	22	USA	Gestational—diet controlled	46	57 ml
**5**	[P5,T2, high]	40+	No	23	Australia	Type 2 diabetes	35	34 ml
**6**	[P6,T1, low]	35–39	Yes	47	Australia	Type 1 diabetes	17	1 ml
**7**	[P7, GDM‐in, high]	30–34	Yes	23	Australia	Gestational—requiring insulin	25	25 ml
**8**	[P8, GDM‐in, low]	30–34	Yes	25	India	Gestational—requiring insulin	24	1 ml
**9**	[P9, GDM, high]	30–34	No	33	Singapore	Gestational—diet controlled	15	55 ml
**10**	[P10,T1, low]	35–39	Yes	39	Australia	Type 1 diabetes	13	Nil

^a^ Participant identifier codes: P = participant number; GDM = gestational diabetes (diet controlled), GDM‐in = gestational diabetes (required insulin), T1 = Type 1 diabetes, T2 = Type 2 diabetes; low = total volume expressed was lower than the median volume expressed in the DAME trial (5.5 ml), high = total volume expressed was higher than the median volume expressed in the DAME trial (5.5 ml).

Analysis of the in‐depth interviews identified six themes: (1) learning and adapting expressing, (2) feelings and sensations associated with expressing, (3) support, (4) dis/empowerment, (5) health, and (6) the value of breast milk. The themes, categories and codes are provided in Table 3.

**Table 3:** Key themes, categories, and codes from in‐depth interviews with participants

Theme	Categories	Codes
Learning and adapting antenatal expressing	Expressing process	Interactions with staff
		Being taught and understanding expressing
	Time for expressing	Finding time for expressing
	Expressing technique	New concept for women
		Finding techniques to suit
	Expressing and labour onset	Linking expressing and labour
	Pump use	Thinking about using pump for expressing
Feelings and sensations associated with expressing	Expressing sensations	Physical sensations associated with expressing
	Feelings about contractions	Possibility of contractions with expressing
	Privacy and awkwardness	Learning expressing felt awkward and needing privacy
Support	Family support	Partner/family support for expressing
	Health professional support	The need for health professional support
	Technique follow‐up	Expressing technique follow‐up after initial teaching
	Cultural and family conflict	Cultural and generational conflict
Dis/empowerment	Empowerment	Confidence
		Achievement
	Disappointment	Disappointment, realism, and acceptance
		Previous or anticipated breastfeeding experience
	Motivation	Diabetes as a motivator
		Wanting to be a good, protective mother
Health	Foetal and baby health	Monitoring, worrying, and feeling guilty about baby when pregnant Worrying about baby after birth
	Maternal health	Adapting work or life for health, diabetes, and pregnancy
	Diabetes	Burden of diabetes
	Worry and stress	Fear and concerns about antenatal expressing
The value of breast milk	Perceptions of milk	Breast milk
		Infant formula
		Expressed breast milk is precious
	Milk volumes	Comparing breast milk volumes to others
		Perceptions of breast milk volumes
		Store of breast milk is security

### 3.1 Learning and adapting antenatal expressing

Five categories were identified under the theme of learning and adapting antenatal expressing: the expressing process, time for expressing, expressing technique, labour onset and expressing, and the possibility of using a breast pump.

#### 3.1.1 Expressing process

Most participants did not know about antenatal expressing before the study and needed an explanation about how expressing milk antenatally ‘worked’, and how to express and store breast milk. The women felt informed and reassured by this information.

*Physically it was nice to be able to have the lactation consultant show you how to do it properly so that at least you felt more informed … Even that sliverest [sic] of information felt, well, “at least I've got this bit”. [P7, GDM‐in, high]*

#### 3.1.2 Time for expressing

Women described managing their diabetes as a significant time burden; checking their blood glucose levels, and managing their insulin and meals made it was difficult for some women to find time to express, particularly for those in paid employment or caring for other children. Some women reduced the length of time they expressed to fit it into their schedule.

*You are feeling uncomfortable and tired and had other little ones, umm it [expressing] was a bit of an extra thing to do. [P1, GDM, low]*

*I was stressed because I had to finish the morning [expressing] course. I definitely found five minutes but not more than that. [P8, GDM‐in, low]*

Alternatively, some women described antenatal expressing was easy to accommodate into their lives.

*It's only 10 min you know, not a big deal really … its only 10 min, twice a day, it's easy. [P4, GDM, high]*

#### 3.1.3 Expressing technique

The instructions and education on expressing gave participants a feeling of ‘being more informed’. Women valued the professionalism and connection with the midwives teaching them but felt that some midwives were not as skilful at teaching hand expressing and therefore adapted the technique to suit themselves.

*You did teach me how to do it on a teaspoon … I found that difficult … I just adapted that [technique] for myself. [P4, GDM, high]*

*Somebody could be great at expressing themselves, but they may not be great at expressing how to – for other people to express. Teaching is a skill. [P6, T1, low]*

#### 3.1.4 Expressing and labour onset

Before consenting to participate in the trial, women were informed of a possibility of earlier birth associated with antenatal expressing (Soltani & Scott, 2012). Some women viewed this as a desirable outcome and thought of using antenatal expressing to induce labour.

*I just thought it was a really good idea, to have it [breast milk] and plus I was keen to bring on labour soon, so I thought it wasn't going to do any harm. [P4, GDM, high]*

#### 3.1.5 Pump use

The intervention taught was hand expressing, however, in the interview some women asked if using a breast pump might be helpful to improve expressing stimulation, to get more milk, ‘help toughen up’ their nipples for breastfeeding and induce labour.

*… hand expressing is difficult but if you can pump to kind of stimulate things is that an option? … hand expressing and squeezing a couple of times and getting some drops out is a bit different to like whacking the pump [on] … also if you're trying to induce labour at 38 weeks then, yeah, why not? Stick it on. [P7, GDM‐in, high]*

### 3.2 Feelings and sensations associated with expressing

Participants were asked what they noticed for themselves or their baby when they were expressing. Three categories were identified: physical sensations during expressing, feelings about any uterine contractions, and privacy and awkwardness.

#### 3.2.1 Expressing sensations

Most women who discussed physical breast sensations associated with expressing referred to minor discomfort to being so uncomfortable or painful that they stopped expressing. For some, the spoon used to collect milk felt awkward and caused nipple soreness.

*… it's [expressing] like squeezing and squeezing and little bit uncomfortable. It's a painful kind of thing. And then you stop it. [P3, T2, low]*

On the other hand, one woman was more positive about the sensations she experienced.

*[physically] It was fine. It was good to try and figure out how to do it properly. [P7, GDM‐in, high]*

#### 3.2.2 Feelings about contractions

Some women felt fearful that the contractions would commence preterm labour, while others hoped the contractions might help them have their baby ‘on time’.

*Sometimes I would feel contractions, sometimes I could feel baby moving and it would obviously evoke fear as well, like oh no, am I starting contractions now or starting some premature labour. [P9, GDM, high]*

*… some yeah contractions [with expressing], some cramping not actually painful or anything, just like you could just feel the tightenings. It wasn't anything major, I never had to stop … Maybe if it was painful then probably, maybe I would have stopped but then I was keen to have him on time, I guess. [P4, GDM, high]*

#### 3.2.3 Privacy and awkwardness

When remembering expressing, women described feeling awkward, uncomfortable and that expressing was a ‘private act’. Having a partner who supported them with expressing was helpful.

*This is a sort of very private act. I don't think I would have been doing it in front of anybody and certainly not anybody that I felt would have been willing to say anything untoward about it. [P6, T1, low]*

*I wasn't doing it the right way and then it was my husband actually who helped me out. It was something new for us and we were just giggling … all mixed feelings, excitement at the same time, a little bit uncomfortable with each other, you know, everything all together. [P3, T2, low]*

### 3.3 Support

The theme of support has four categories: appreciating family support, wanting health professional support, needing professional follow‐up about their expressing technique, and expressing as a potential source of conflict.

#### 3.3.1 Family support

The women appreciated the physical and emotional support of their partners and/or close family.

*My partner was really supportive. He went and would fetch me spoons and syringes and stuff and help me label things. [P5, T2, high]*

#### 3.3.2 Health professional support

In the trial, the women were supervised during their first expressing and were encouraged to contact the research team if they had questions or concerns when expressing at home. The women reflected that the knowledgeable support from the health professionals made them feel comfortable and was highly valued.

*It was good to try and figure out how to do it [expressing] properly … They were very professional and made me feel comfortable in doing that because going through pregnancy and childbirth and all, that it's – the first time it's very exposing and she was very–made me feel very comfortable and was comforting, I suppose … I think that if the interaction was negative that I wouldn't have persisted with it or wouldn't have engaged with it. [P7, GDM‐in, high]*

#### 3.3.3 Technique follow‐up

Some women, especially primiparous women, were uncertain if their expressing technique was ‘right’. Participants suggested that a scheduled follow‐up from a midwife or lactation consultant would have been helpful.

*Then as a follow‐up when you have your next clinic appointment that someone could check your technique. Just to double check and to give you that confidence that things are going well and that you are getting it right. I guess it's just that confidence to know that you are doing it right. [P10, T1, low]*

#### 3.3.4 Cultural and family conflict

If antenatal expressing was unfamiliar to a woman's family member, who subsequently voiced their concerns to the participant, expressing became a source of conflict.

*It was just my mum, she is like ‘What is this? Why don't we have this in Singapore?’ … I was educating her – all babies' tummy, first day, is just the size of cherry. This is trying to educate her when the conflict arose about the antenatal expressing. [P9, GDM, high]*

### 3.4 Dis/empowerment

Three categories were identified in the theme of dis/empowerment: empowerment, disappointment, and motivation.

#### 3.4.1 Empowerment

Antenatal expressing was identified by some participants as improving their breastfeeding motivation and confidence and gave a sense of achievement and control in a pregnancy that was ‘controlled by diabetes’.

*The fact that then I was able to express something and save something it was kind of an accomplishment. It felt like something I could do to empower myself and assist my child because everything else felt like it was taken out of my hands. [P7, GDM‐in, high]*

#### 3.4.2 Disappointment

While care was taken to inform women that expressing little or no milk antenatally is probably normal, some women with low or no milk described feeling stressed and disappointed, and a sense that their bodies had not functioned as they had hoped.

*It's not really disappointing but kind of. Sometimes I feel a bit low that I can't express. [P8, GDM‐in, low]*

#### 3.4.3 Motivation

For some women, the anticipation of breastfeeding problems or the ability to express milk was motivation to continue expressing. However, others felt expressing was futile when they were unable to express any milk.

*By participating and hand expressing and getting something, kind of encouraged me to keep going. [P7, GDM‐in, high]*

*You do have that sense that, is there any point in me doing this? … if it had a been more successful with getting milk, I'm sure that it would have pushed me on a bit more. [P1, GDM, low]*

### 3.5 Health

The theme of health has four categories: foetal and baby health, maternal health, diabetes, and worry and stress for the women.

#### 3.5.1 Foetal and baby health

Participants described general concern for their babies but particularly their fears of macrosomia, neonatal hypoglycaemia, separation from their infant, and breastfeeding difficulties.

*I was always worrying about whether she was going to be a giant baby. [P1, GDM, low]*

*I was concerned about how my blood sugars were going to be affecting my unborn child. [P6, T1, low]*

#### 3.5.2 Maternal health

The women's own health, especially their diabetes, became a major focus during their pregnancy. Advice from some health professionals made women feel uncomfortable and guilty for having diabetes, especially if a woman was above her ideal weight. Women felt a responsibility to be a ‘good’ mother by caring for their own health and by doing so, the health of their foetus and baby.

*About the blood sugars and the baby – the risk to the baby. Like “you have gestational diabetes. You are a risk. Even your baby's life. With your blood”. That was the attitude that I experienced from them [hospital staff]. … That was my feeling of that's their priority not anything else … I suppose my prioritisation, my whole pregnancy became about my diabetes. It wasn't about anything else in my pregnancy. [P7, GDM‐in, high]*

#### 3.5.3 Diabetes

The diagnosis of diabetes was a shock for some women. Their health became a priority and they needed to adapt their work and home life for regular blood glucose monitoring, eating and exercise. Their diabetes management was considered the major burden in their lives.

*I was actually shocked when I got a call from the hospital saying that [I] had gestational diabetes … You have to explain to a lot of people what you are doing and why you are doing it because you're expecting so you have to take care of the baby and yourself. [P8, GDM‐in, low]*

*I didn't feel that [expressing] was difficult, maybe because I was already put on this after having to take blood sugar levels four times a day. That was even more difficult than the expressing. [P9, GDM, high]*

#### 3.5.4 Worry and stress

Some women and their families were concerned that expressing might be taking nutrients from their foetus, or that breast milk would be ‘spoilt’ with freezing.

*As we know, normally milk do get, like, spoiled. That was one of the concerns of my mother but when it's frozen, it's frozen. Like, it shouldn't get rot? [P8, GDM‐in, low]*

*What if I'm taking nutrients from him, [with expressing] I kind of panicked from that perspective? [P4, GDM, high]*

### 3.6 The value of breast milk

Two categories were identified: perceptions of milk, and milk volumes.

#### 3.6.1 Perceptions of milk

Women described their breast milk as ‘valuable’, ‘precious’, ‘liquid gold’, and ‘natural’, and wanted to avoid the use of infant formula where possible. Women were disappointed if their breast milk and therefore, expressing efforts were wasted. Participants questioned if staff, who the women believed should know the value of breast milk, actually valued their breast milk as highly as they did.

*I felt like all that work to get all that milk and most of it got wasted unfortunately, just from being left out and being forgotten about … so, when I came in the next morning, it was just sitting under there [cot] and they hadn't got any more out. And they had just given him [infant] formula … [P5, T2, high]*

#### 3.6.2 Milk volumes

Women were aware of the possibility of postpartum problems associated with maternal diabetes such as low milk production and low infant blood glucose levels, so a store of breast milk was considered as security. Yet some wondered if their small amounts of breast milk would be enough.

*I understand that with gestational diabetes they might come out hypo and need the milk instantly. So, I want it to have a little bit of stash for him or her just in case I didn't have any [breast milk] … [P9, GDM, high]*

*I thought it was such small amounts, is it going to make any difference? [P5, T2, high]*

When asked if they would express again, all participants said they would try, but that they would stop if they could not express any milk. Some had been encouraging pregnant family members or friends to express antenatally, and not just those with diabetes. Regardless of the volume they had obtained, participants felt overall the experience of antenatal expressing was positive, as it had provided stimulation, and they hoped for better milk volumes if they expressed again.

*For anybody that's not getting any milk, to be like ‘keep it up cos you know cos these are the benefits of just the stimulation’. [P1, GDM, low]*

*It might be something that if you want to try this, you can, but don't expect too much. [P5, T2, high]*

## 4 DISCUSSION

This study, which explored the views and experiences of antenatal expressing for women with diabetes in pregnancy, has confirmed similar themes in previous studies and identified new themes. Consistent with previous studies we found that antenatal expressing gave some women feelings of confidence, achievement, and empowerment (Brisbane & Giglia, 2015; Casey, Mogg, et al., 2019; Demirci et al., 2019) but that expressing at times felt uncomfortable, painful (Demirci et al., 2019) and embarrassing (Brisbane & Giglia, 2015; Demirci et al., 2019). Also consistent with earlier studies, women wanting to avoid infant formula, saw their milk as precious and security for their baby's health, and that by expressing milk they were being a good and protective mother (Casey, Mogg, et al., 2019). Similar to findings by Casey, Mogg, et al. (2019), and Brisbane and Giglia (2015), women in this study described feelings of frustration, disappointment, and futility if their breast milk was not used, or hospital staff did not value their precious breast milk as much as they did. Some women wondered if antenatal expressing was worth their efforts.

Difficulty in finding time to express was commonly mentioned in the routine follow‐up in the DAME trial and by Demirci et al. (2019), so was included in the interview guide. Some of the participants had expressed antenatally again since the trial and described finding time to express was more difficult now that they were caring for other children and said they ‘did it when they could’.

Due to increased pregnancy monitoring for women with diabetes, some participants described a loss of control and that managing their diabetes felt burdensome. Yet for some, the addition of twice‐daily expressing did not increase their overall feelings of burden. Antenatal expressing created some positive feelings. Some women felt that expressing gave them a level of control in their pregnancy, in that they might be able to produce a store of breast milk for their baby and hoped that antenatal expressing might improve their approaching breastfeeding experience.

For some women, including their partner in the expressing experience was positive, with their partners encouraging them, helping with equipment and hands‐on expressing. However, others described conflict when other family members questioned their antenatal expressing. As with the evidence to support breastfeeding overall (McFadden et al., 2017), the inclusion of partners or support people when teaching antenatal expressing may support women and reduce familial conflict.

Our study further confirmed that women wanted support from health professionals at the time of initial expressing education and at subsequent antenatal appointments. Given that they had only had one supervised expressing session, they wanted to know if they were ‘doing it right’. Demirci et al. (2019) discussed that concerns about breast tenderness, expressing technique, milk volumes or contractions may be addressed with observation, feedback, and knowledgeable discussions. Our participants confirmed they would find follow‐up useful and acceptable.

Participants discussed feelings of empowerment, confidence, sense of control, and the possibility of increased exclusivity of breast milk that antenatal expressing provided, but also acknowledged feelings of futility, pain, and fear. The women in this study were considered low risk for pregnancy concerns other than their diabetes, so the views and experiences of antenatal expressing women for experiencing higher risk pregnancies, or without diabetes may be different.

Participants stated they would express antenatally again, but only if they were getting milk. This study found that some women question the effectiveness of advising antenatal expressing for all women, and if there are there ‘real benefits’ for their efforts of expressing. Women with diabetes in pregnancy are currently the target group being advised to express antenatally, and the management of diabetes in pregnancy may be a significant time and energy burden, so the prospect of antenatal expressing little or no breast milk may feel too onerous and stressful for some women. When advising women to express antenatally, health professionals should discuss with women the potential additional burden versus realistic potential benefit, especially in the context of her diabetes and other caring or work roles.

Consistent with the studies by Demirci et al. (2019) and Casey, Mogg, et al. (2019) our study highlighted women's disappointment and frustration with hospitals that are advising antenatal expressing, yet not consistently supporting the storage and prioritised use of their precious breast milk. Given the substantial efforts women put into expressing, maternity care providers need to ensure training for staff about antenatal expressing and proactive support for women who are expressing antenatally. Hospitals must also ensure the clear identification and safe storage of antenatal breast milk and prioritise its use when required. With the increased awareness and use of breast pumps generally, health professionals should also be alert that pregnant women may be considering using a breast pump to express antenatally, which is yet to be researched.

A limitation of this study is the small sample of participants from the intervention arm of a randomised‐controlled trial. Two of the participants interviewed were taught antenatal expressing at randomisation by the first author and it is possible that these participants may have modified their responses. To what extent the participants modified their responses remains unknown. Some of the themes may have been also identified by participants in the control arm—especially relating to the values of importance of breast milk, concern about breastfeeding in general, and the burden of diabetes. While diabetes in pregnancy was not the focus of this study, the women's diabetes management had a significant day‐to‐day impact and was often woven into participants' responses about expressing. Also, the interviews were conducted approximately 3 years after the women had participated in the trial, however research has confirmed that women's memory of infant feeding is accurate up to at least 6 years postpartum (Amissah et al., 2017). A strength of this study is that women who have diabetes in pregnancy are currently a target group to be advised to express antenatally and these in‐depth interviews have provided valuable insights into their lives, views, and experiences.

This is the first study describing the views and experiences of women expressing antenatally with the researchers knowing the volumes of antenatal breast milk. Currently there is no evidence that antenatal expressing promotes increased postnatal volumes of breast milk. A perceived lack of a sufficient milk supply is often the main reason cited by women for ceasing breastfeeding earlier than their intended goals (Morrison et al., 2015), so health professionals should be alert to women being concerned if they express little or no milk and be ready to reassure them.

While research is emerging about antenatal expressing, other evidence‐based breastfeeding interventions must be maintained, such as the global initiative of the BFHI Ten Steps to Successful Breastfeeding (World Health Organization, 2017), particularly the need for health professionals to discuss the importance and management of breastfeeding with pregnant women and their families. This will ensure the intervention of antenatal expressing complements, but does not overshadow established best practices that protect, promote, and support breastfeeding.

## CONFLICT OF INTERESTS

Della A. Forster, Lisa H. Amir, and Anita M. Moorhead report a grant from the Australian National Health and Medical Research Council, for the conduct of the study.

## AUTHOR CONTRIBUTIONS

AMM, LHA, DAF designed the research study. AMM identified participants and conducted the interviews. AMM, SBC and LHA analysed the data. Anita M. Moorhead drafted the manuscript. AMM, LHA, DAF and SBC finalised the manuscript.

## Data Availability

The data that support the findings of this study are available on request from the corresponding author. The data are not publicly available due to privacy and ethical restrictions.
